# Rates and predictors of stroke-associated case fatality in black Central African patients

**Published:** 2008-04

**Authors:** B Longo-Mbenza, J Mbuilu Pukuta, M Lelo Tshinkwela

**Affiliations:** Department of Cardiology and Pathophysiology, Division of Cardiology, University of Kinshasa, Kinshasa, Democratic Republic of Congo; Department of Cardiology and Pathophysiology, Division of Cardiology, University of Kinshasa, Kinshasa, Democratic Republic of Congo; Computer Tomography Scan Unit, Division of Radiology, University of Kinshasa, Kinshasa, Democratic Republic of Congo

## Abstract

**Objective:**

To identify case fatality rates and predictors of stroke in a private clinic in Kinshasa, Democratic Republic of Congo.

**Methods:**

Two hundred and twelve black Africans were consecutively admitted to a clinic and prospectively assessed during the first 30 days by CT scan-proven stroke types and outcome. Univariate and multivariate analyses were used to estimate the in-hospital mortality risk for the following baseline characteristics: age, gender, education, arterial hypertension, diabetes, stroke types, leukocyte count, and haematocrit, blood glucose, uric acid, fibrinogen and total cholesterol levels.

**Results:**

Haemorrhagic and ischaemic strokes were present in 52 and 48% of the study population, respectively; and 44% of all stroke type patients, 29% of haemorrhagic stroke and 31% of ischaemic stroke patients died. Compared to the survivors, deceased patients were significantly (*p* < 0.001) older with higher leukocyte counts and haematocrit, haemoglobin and fibrinogen levels, but lower glycaemic levels. The variable significantly associated with all stroke type mortalities in the multivariate model was ischaemic stroke (HR = 4.28, *p* < 0.001). The univariate risk factors of mortality in patients with ischaemic stroke were higher fibrinogenaemia (RR = 6.4; 95% CI = 4.8−8.2 for tertile 3 and RR = 12.9; 95% CI = 7.8–18.4 for tertile 4; *p* < 0.001) and higher glycaemia (RR = 3.3; 95% CI = 1.4–5.7 for tertile 3 and RR = 6.7; 95% CI = 5.2–9.2 for tertile 4; *p* < 0.001).

**Conclusion:**

We have shown that all acute stroke types remain a deadly nosological entity, and ischaemic stroke, baseline haematocrit and fibrinogen levels, and dependency on others’ care were significantly associated with all stroke mortalities. Moreover, hyperfibrinogaemia and hyperglycaemia were the significant predictors of case fatality in ischaemic stroke patients. In Africa, the top priority for resource allocation for stroke services should go to the primary prevention of stroke.

## Summary

Stroke is the third commonest cause of mortality in western industrialised countries^1^,^2^ and among African–Americans.^3^ The adequate management and control of hypertension and other risk factors^4^,^5^ have contributed to the decline in stroke incidence and mortality in developed countries since the 1940s, with accelerated decreases from the late 1960s.^6^

The profile of morbidity and mortality observed in most developing countries in Africa, such as the Democratic Republic of the Congo (DRC),^7^,^8^ is similar to that of western societies before the 20th century. Stroke is therefore no longer thought to be a rare diagnostic curiosity in black Africans.

Arterial hypertension and its complications (congestive heart disease, cerebrovascular events and end-stage renal disease) were found to be the second cause of morbidity in patients hospitalised at the hospital of Kinshasa University, exceeded only by liver diseases^7^ or infectious diseases.^8^ Stroke accounted for 12% of the overall mortality and for 57% of cardiovascular deaths.^8^ In a prospective population-based study of black residents of Harare, Zimbabwe, with a standardised incidence rate of 68 per 100 000 and a first-week mortality rate of 35%, stroke was also considered an important cause of morbidity and mortality in the African community.^9^ The epidemiology of stroke in Africans is now better understood.^10^-^23^

In a recent clinical and epidemiological study at the University Hospital of Kinshasa, we reported that blood glucose and urea levels and leukocyte counts were higher in patients who died than in survivors, and altered awareness of risks for stroke was the major independent predictor of fatality rates.^10^ However, there is no information on the association between total cholesterol and triglyceride levels, stroke types and stroke-related case fatalities in a population with favourable lipid profiles. Our study addressed this aspect, with the focus on non-academic hospital mortality rates and predictors.

## Methods

This short prospective study was carried out at the Lomo Medical Centre, Kinshasa-Limete, DRC between 1989 and 1992. The Lomo Medical Centre is a non-academic hospital specialising in ultrasound and Doppler diagnosis for the care of patients with cardiovascular diseases. It has an eight-bed emergency room and is located in the central inner quarter of Kinshasa. The capital of DRC, located at latitude 4° 20'S, longitude 15°16'E, and altitude 358 m, Kinshasa is a huge tropical city of six million people with a low standard of living and no easily accessible and affordable heathcare. A large number of primary-care centres, private or under the auspices of the churches and the Congolese state, refer cases of stroke to Lomo Medical Centre for cardiovascular investigation and hospitalisation for specialised management.

Included in the study were all stroke patients admitted within the first 72 hours after onset of symptoms. Patients with transient ischaemic accident and those with subarachoid haemorrhage were excluded from the study. The study protocol was approved by the ethics committee of the Lomo Medical Centre.

In total, 212 patients were included in the study and either gave their consent themselves, or the relatives of comatose and aphasic patients gave the necessary consent. For each patient, age, gender, education level, medical history and outcome were recorded. Stroke criteria were defined according to the WHO criteria.^11^ Blood samples for the determination of blood glucose, haemoglobin, haematocrit, triglycides, leukocyte count, urea, creatinine, uric acid, fibrinogen and total cholesterol levels were collected on admission and were considered potential predictors.

The rates of hypertension and diabetes mellitus were obtained from the medical history and clinical investigations. Non-invasive blood pressure measurements were taken using a standard sphygmomanometer and auscultation, with Korotkoff phase I as systolic and phase V as diastolic blood pressure. Hypertension was diagnosed in patients with systolic blood pressure ≥ 140 mmHg or diastolic blood pressure ≥ 90 mmHg or both, and a history of hypertension, reported by general physicians or relatives. Diabetes mellitus was diagnosed on the basis of complete overnight fasting plasma glucose concentrations ≥ 126 mg/dl and/or a history of diabetes and current treatment with hypoglycaemic agents.

Computed tomography (CT scan) of the brain was performed routinely for all 212 patients within 72 hours of admission at the University Kinshasa Hospital (Dr Lelo T) as described in detail elsewhere.^13^ The distinction between haemorrhagic and ischaemic stroke types was based on the CT scan.

Details of each patient’s demographic data, biological parameters, presenting complaints, neurological examination and outcome until discharge were prospectively recorded in a daily ward surveillance and transferred to a computerised database.

## Statistical analysis

Data were expressed as means ± standard deviation (SD) and proportions (%). The group differences were evaluated with the Student’s *t*-test for continuous variables and with the Chi-squared test for categorical data.

To assess the independent effects of baseline risk factors associated with in-hospital stroke mortality, a Cox proportional hazards model was performed with SPSS version 11. The variables entered into the multivariate model were age, gender, education level, history of hypertension and diabetes, stroke type, and baseline levels of haematocrit, blood glucose, uric acid, total cholesterol, fibrinogen and leukocyte count.

Stroke types were then excluded in order to perform the second multivariate model with all the remaining baseline variables. Risk (HR = hazards ratio) of stroke mortality and associated 95% CI (confidence interval) were calculated from the regression coefficients. Probability of less than 0.05 was considered significant.

## Results

The rates of hypertension and diabetes mellitus among the stroke patients were 81% (*n* = 172) and 14.6% (*n* = 31), respectively. Over 94.2% of the hypertensive patients were not taking antihypertension medication before the onset of stroke. Haemorrhagic stroke was present in 110 patient (52%) and ischaemic stroke in 102 patients (48%).

During the surveillance period, 94 of 212 all-stroke patients (44%), 62 of 110 haemorrhagic stroke patients (29%) and 32 of 102 ischaemic stroke patients (31%) died. Baseline characteristics of the survivors compared to those of the deceased patients are presented according to stroke type in Tables 1 and 2. Deceased patients were older (*p* < 0.001) in both stroke categories. Haematocrit, haemoglobin, leukocyte count and fibrinogen levels were higher (*p* < 0.001) in all deceased haemorrhagic and ischaemic strokes patients.

**Table 1.** Comparison Of Age And Baseline Haematologic Parameters According To Stroke Types And Outcome

*Variable and stroke type*	*Study population*	*Fatal stroke*	*Non-fatal stroke*	*p*
All stroke types				
Age (years)	57.8 ± 10.9	61 ± 10	55 ± 11	< 0.001
Haemoglobin (g/dl)	14 ± 2	15 ± 2	13 ± 1	< 0.001
Haematocrit (%)	41 ± 7	46 ± 8	38 ± 4	< 0.001
Leucocyte (10^3^/mm^3^)	7.8 ± 3.7	9.5 × 10^3^ ± 3.4 × 40^3^	6.4 × 10^3^ ± 3.4 × 10^3^	< 0.001
Fibrinogen (mmol/l)	12.6 ± 4.3	15 ± 4	11 ± 4	< 0.001
Haemorrhagic stroke				
Age		60.6 ± 10.3	55 ± 9	< 0.001
Haemoglobin		16 ± 2	13 ± 1	< 0.001
Haematocrit		46 ± 8	38 ± 4	< 0.001
Leucocyte		10 × 10^3^ ± 3 × 10^3^	6.6 × 10^3^ ± 3.6 × 10^3^	< 0.001
Fibrinogen		15 ± 4	10 ± 3	< 0.001
Ischaemic stroke				
Age		63 ± 10	55 ± 12	< 0.001
Haemoglobin		15 ± 2	13 ± 1	< 0.001
Haematocrit		45 ± 8	38 ± 5	< 0.001
Leucocyte		9 × 10^3^ ± 3 × 10^3^	6 × 10^3^ ± 3 × 10^3^	< 0.001
Fibrinogen		14 ± 4	12 ± 4	< 0.005

**Table 2.** Comparison Of Baseline Metabolic Parameters According To Stroke Type And Outcome

*Variable and stroke type*	*Study population*	*Fatal stroke*	*Non-fatal stroke*	*p*
All stroke types				
Glucose (mmol/l)	7 ± 6	6.2 ± 3	7 ± 6.8	< 0.001
Urea (mmol/l)	5 ± 3	5 ± 15	4.8 ± 3.5	< 0.001
Creatinine (μmol/l)	91 ± 39	91 ± 51	85 ± 65	< 0.001
Uric acid (μmol/l)	369 ± 149	393 ± 137	357 ± 179	< 0.001
Total cholesterol (mmol/l)	5.2 ± 0.1	5.3 ± 1.2	5.1 ± 1.3	< 0.001
Triglycerides (mmol/l)	1.4 ± 0.7	1.6 ± 0.8	1.4 ± 0.7	< 0.001
Haemorrhagic stroke				
Glucose (mmol/l)		6.5 ± 2.7	8.5 ± 10	< 0.005
Urea (mmol/l)		5.2 ± 1.6	4.9 ± 3.7	< 0.001
Creatinine (μmol/l)		97.5 ± 33	76 ± 98	< 0.001
Uric acid (μmol/l)		417 ± 113	357 ± 179	< 0.001
Total cholesterol (mmol/l)		5.2 ± 1.2	4.9 ± 1.1	< 0.001
Triglycerides (mmol/l)		1.6 ± 0.7	1.2 ± 0.6	< 0.001
Ischaemic stroke				
Glucose (mmol/l)		7 ± 3.6	6.8 ± 4.6	< 0.001
Urea (mmol/l)		4.7 ± 1.5	4.8 ± 3.5	< 0.001
Creatinine (μmol/l)		85 ± 26	78 ± 35	< 0.001
Uric acid (μmol/l)		381 ± 179	357 ± 115	< 0.001
Total cholesterol (mmol/l)		5.3 ± 1.4	5.4 ± 1.4	< 0.001
Triglycerides (mmol/l)		1.7 ± 0.8	1.5 ± 0.7	< 0.001

Serum creatinine, urea and uric acid levels were elevated (*p* < 0.05 and *p* < 0.001) in all-stroke and haemorrhagic stroke patients, but lower in surviving and deceased ischaemic stroke patients. Blood glucose levels were lower in fatal all-stroke and non-fatal haemorrhagic stroke patients than in other stroke patients, but similar in both deceased and surviving ischaemic stroke patients. Except that the highest (*p* < 0.01) levels of serum triglycerides were observed in the deceased haemorrhagic stroke patients, the profiles of total serum cholesterol in each stroke type and triglycerides in all-stroke and ischaemic stroke patients were similar in both deceased and surviving patients. Fifty per cent (*n* = 15) of diabetics and 49% (*n* = 30/60) of patients with hyperglycaemia after all-stroke and ischaemic stroke died, respectively.

In the multivariate analysis and compared to haemorrhagic stroke, the ischaemic stroke type was significantly (HR = 4.28, 95% CI = 1.38−13.2; *p* < 0.001) and positively associated with in-hospital mortality. However, in post hoc and separate univariate analyses and in comparison with tertiles 1, tertiles 2 and 3, plasma fibrinogen (RR = 6.4, 95% CI = 4.8−8.2 and RR = 12.9, 95% CI = 7.8−18.4) and blood glucose (RR = 3.3, 95% CI = 1.4−5.7 and RR = 6.7, 95% CI = 5.2−9.2) levels were the significant (*p* < 0.001) predictors of mortality in ischaemic stroke patients but not in all haemorrhagic stroke types.

## Discussion

The present study results are consistent with rare hospital-based statistics^7^,^8^,^10^ and a population-based survey^9^ that have recognised stroke as a deadly and emerging disease among the leading causes of cardiovascular deaths for black Africans. The observed case fatality of 44% in this private clinic-based study was similar to that reported (44%) recently in the same city from a public hospital,^10^ but higher than that reported (35%) in Harare, Zimbabwe, from a community-based surveillance.^9^ These results suggest that stroke remains a deadly nosological entity in black Africans^14^ as well as in Africa−Americans.^15^

Using multivariate analyses, ischaemic stroke emerged as the significant predictor of in-clinic mortality in a private clinic, with a four-fold higher risk than haemorrhagic stroke. This indicates that the clinical spectrum of stroke is changing over time among Africans. The higher rates of haemorrhagic stroke (52%), previously reported in Africans,^21^,^22^ than in the present study, based partly or entirely on clinical criteria, could be explained by severe, uncontrolled hypertension. Indeed, our recent report on stroke with the diagnosis of stroke types defined using brain CT scan revealed a ratio of one haemorrhagic stroke (*n* = 55) to two ischaemic strokes (*n* = 99). In black Africans, the prevalence of ischaemic stroke rises with advancing age, whereas rates of haemorrhagic stroke decrease with age.^23^ Advancing age may also promote the effects of other atherosclerotic factors such as dyslipidaemia, higher blood viscosity (haematocrit, fibrinogen), smoking, obesity and so on.

Increased blood viscosity was found in the deceased stroke patients (highest levels of haematocrit and fibrinogen observed) and may indicate one mechanism that promotes all major risk factors of cardiovascular disease.^24^ Several studies have shown that haematocrit^25^,^26^ and fibrinogen^27^ levels are predictive for risk of stroke. Indeed, in this study, the risk of mortality was multiplied by six and 13 in ischaemic stroke patients with plasma fibrinogen levels of 350–452 mg/dl and 453–760 mg/dl, respectively, compared with those admitted with plasma fibrinogen below 350 mg/dl.

In a large consecutive series of 1 032 urban Congolese patients admitted to the University of Kinshasa Hospital,^28^ we found that haematocrit was significantly correlated with plasma fibrinogen levels, and the mortality risk for individuals with haematocrit above 40% was six-fold greater than that of individuals with a haematocrit below 40%. Dehydration is common in the hot tropical climate of Kinshasa and this may also reflect inappropriate fluid and feeding levels in comatose patients.

The negative association, although weakly significant, between stroke mortality and blood glucose levels as well diabetes mellitus in this study using multivariate analysis was not obvious.^29^,^30^ The present findings were not in agreement with those of our previous study undertaken in a public hospital,^10^ which did demonstrate a positive and significant association of diabetes mellitus and hyperglycaemia with acute stroke, as has been reported in developed countries. By contrast, in the present study, a *post hoc* and univariate separate logistic regression identified a three- and seven-fold increased risk of mortality for ischaemic stroke patients with tertiles 3 and 4 of blood glucose in comparison with those in tertile 1 of blood glucose. Studying cardiovascular mortality in Congolese diabetics, Bafende^31^ reported that a negative and significant association between blood glucose and blood pressure as well as between blood glucose and age, fitted a multivariate analysis that isolated arterial hypertension as the only significant and independent determinant of cardiovascular morbidity.

The negative influence of hyperglycaemia on blood pressure and stroke mortality may be explained by the frequent end-stage renal impairment seen in hypertension and diabetes mellitus, which determines associated hyperinsulinaemia, higher blood pressure and lower glycaemia.^32^ Furthermore, the present study showed that stroke survivors had higher baseline levels of blood glucose than patients with fatal strokes.

Similar levels of total serum cholesterol and triglycerides in deceased and surviving stroke patients may explain the absence of association between stroke mortality and lipid profiles. The association between stroke, mortality and lipid levels is not clear in Africans with low risk of coronary heart disease and low total cholesterol.^7^ Hyperlipidaemia has not been found to contribute to the pathogenesis of stroke in Africans.^33^

This study was limited to some extent in that it was not undertaken among the general population. However, as more than 95% of patients with acute stroke are hospitalised in this urban area of Kinshasa, major sampling bias was considered to be unlikely. Other limitations were due to the difficulties faced in a developing country, such as, likely selection bias due to care/deaths at home, referral patterns, case selection, missing data, and information bias due to errors in diagnosis or risk factor information.

Available literature on the epidemiology of stroke in Africa is limited. For that reason, many factors influencing stroke, such as HIV/AIDS, syphilis, sickle cell disease, thromboembolism, arrhythmia, insulin resistance, and abnormalities of the rennin angiotensin system, serum adiponectin and plasma aldosterone, were not studied. The demography and poverty of Africans contribute to the lack of complete data, accuracy of diagnosis and the representativeness of the study population within clinics. 34 Nevertheless, our hospital-based data probably represent the situation in the African population in general because all acute medical cases are admitted to tertiary hospitals.^9^

The accuracy of diagnosis of stroke type (ischaemic or haemorrhagic) was excellent in the present study, with brain CT scan performed in all individuals whose African cultural taboos imposed social inhibitions on the carrying out of *post mortem* examinations to accurately certify the cause of death.^35^

This study reflects, by and large, the types of stroke deaths reported in the private sector, where the problem of accurate mortality statistics poses some limitations on the quality of data.^35^ Moreover, the possible bias related to the use of only clinical criteria was avoided. The study was limited to some degree by not considering smoking and alcohol intake in the interpretation of the influence of haematocrit and fibrinogen on stroke mortality. In the Framingham study, high haemoglobin levels, which are significantly associated with haematocrit, were positively related to an increased risk of stroke, whereas the significant relationship vanished after adjustment for smoking and hypertension.^36^

Since almost all stroke patients included in this study were hypertensives and died, it would have been senseless to enter hypertension and blood pressure in the logistic regression model that failed to outline the role of hypertension in stroke mortality. It has been demonstrated that elevated systolic blood pressure above 160 mmHg or its decrease below 140 mmHg were about three and two times more likely to cause stroke mortality,^37^ while from our previous studies, systolic blood pressure within the range 160–199 mmHg appeared to be optimal for survival^10^ in blacks.

In general, age and hypertension are the most important risk factors for incidence of stroke in the developing world.^34^ Other factors such a diabetes mellitus and high lipid levels, which are less important in stroke than in heart disease in these developing countries,^38^ were not significant predictors of mortality in the African stroke patients in the present study.

## Conclusion

Baseline age, leukocyte count, haematocrit, and blood glucose, haemoglobin and plasma fibrinogen levels were higher in subsequently fatal all-stroke patients than in non-fatal cases. Ischaemic stroke was the significant independent predictor of stroke case fatality. Higher fibrinogenaemia and hyperglycaemia were the risk factors of case fatality in patients with ischaemic stroke.
